# Effects of Jianpi Bushen Therapy for Treatment of CKD Anemia: A Meta-Analysis of Randomized Controlled Trials

**DOI:** 10.3389/fphar.2020.560920

**Published:** 2020-09-15

**Authors:** Liang Li, Chengyin Li, Yu Zhou, Qi Xu, Zilin Wang, Xiaoyun Zhu, Yuanming Ba

**Affiliations:** ^1^ Department of Nephropathy, Hubei Provincial Hospital of TCM, Wuhan, China; ^2^ Clinical College of Chinese Medicine, Hubei University of Chinese Medicine, Wuhan, China; ^3^ Department of Oncology, Hubei Provincial Hospital of TCM, Wuhan, China

**Keywords:** chronic kidney disease anemia, Jianpi Bushen therapy, meta-analysis, randomized controlled trials (RCT), Traditional Chinese Medicine

## Abstract

**Objectives:**

To evaluate the efficacy of Traditional Chinese Medicine, specifically Jianpi Bushen (JPBS) therapy, for treatment of patients with chronic kidney disease (CKD) anemia.

**Methods:**

Randomized controlled trials of JPBS therapy for CKD anemia were searched and selected from seven electronic databases. The Cochrane collaboration tool was used to conduct methodological quality assessment. RevMan v5.3 software was utilized to perform data analysis.

**Results:**

In total, 12 randomized controlled trials with 799 patients met the meta-analysis criteria. The aggregated results indicated that JPBS therapy is beneficial for CKD anemia by improving the clinical efficacy rate [risk ratio (RR) = 1.23, 95% confidence interval (CI): (1.14, 1.33), *P* < 0.00001] and hemoglobin (Hb) [weighted mean difference (WMD) = 9.55, 95% CI: (7.97, 11.14), *P* < 0.00001], serum ferritin (SF) [WMD = 6.22, 95% CI: (2.65, 9.79), *P* = 0.0006], red blood cell (RBC) [WMD = 0.31, 95% CI: (0.24, 0.38), *P* < 0.00001], hematocrit (HCT) [WMD = 2.95, 95% CI: (2.36, 3.54), *P* < 0.00001], serum creatinine (SCr) [WMD = 64.57, 95% CI: (33.51, 95.64), *P* < 0.0001], and blood urea nitrogen (BUN) levels [WMD = 3.76, 95% CI: (2.21, 5.31), *P <*0.00001]. Furthermore, evidence suggests that JPBS therapy is safe and does not increase adverse reactions compared with western medicine (WM) alone.

**Conclusion:**

This study found that JPBS therapy has a positive effect on the treatment of CKD anemia. However, more well-designed, double-blind, large-scale randomized controlled trials are needed to assess the efficacy of JPBS therapy in the treatment of CKD anemic patients.

## Introduction

Anemia is one of the most common complications in patients with chronic kidney disease (CKD), and its prevalence progressively increases as the glomerular filtration rate declines (Hsu et al., 2002; Li et al., 2016). Chronic kidney disease anemia is associated with increased risk of reduced quality of life, cardiovascular disease, cognitive impairment, and mortality (Hörl, 2013). One of the main causes of CKD anemia is a decrease in the renal production of erythropoietin (EPO). In addition, iron, which is an important raw material for hemoglobin production, is often deficient in CKD patients due to impaired dietary iron absorption and increased iron loss (Babitt and Lin, 2012; Gafter-Gvili et al., 2019). Thus, erythropoiesis-stimulating agent (ESA) administration and iron supplementation are the primary Western medicine (WM) treatments for CKD patients with anemia. Although they are effective drugs that clearly avoid the need of blood transfusions, the controversy and safety issues have been raised for both drugs in recent years, especially when they are given at high doses. Evidence has shown that intravenous iron contributes to cardiovascular morbidity and mortality in patients with end-stage renal disease (ESRD) (Kuragano et al., 2014; Bailie et al., 2015). Treatment with ESAs has also been linked to stroke, thrombotic events, and hypertension in CKD patients (Koulouridis et al., 2013). Moreover, 5–10% of patients exhibit an inadequate response to ESAs (Costa et al., 2008), resulting in an increased risk of faster progression to ESRD and all-cause mortality (Solomon et al., 2010; Minutolo et al., 2012). Current WM treatment is also only designed to increase hemoglobin production, not necessarily to improve renal function. The production of EPO will decline with the decrease in glomerular filtration rate and progress of CKD, thereby worsening patient anemia (Prasad-Reddy et al., 2017). Hence, faced with the limitations of current treatment, complementary and/or alternative medicines that improve renal function and alleviate anemia are urgently needed.

Chinese herbal medicine (CHM) has been widely used to treat anemia (Bradley et al., 1999; Chen et al., 2018). According to Traditional Chinese Medicine (TCM) theory, blood deficiency is the main feature of anemia and spleen and kidney deficiencies are the basic pathological features. Based on the relationship between the spleen and kidney and blood generation, classic TCM therapy for the treatment of anemia involves invigorating the spleen and reinforcing the kidney (known as Jianpi Bushen, JPBS). For instance, Wu et al. (2016) found that treatments based on kidney support had a positive effect on treating chronic aplastic anemia. Lin et al. (1990) also found the therapy of warming and tonifying the spleen and kidney improve hemopoietic function of chronic aplastic anemia patients. In recent decades, several clinical trials have evaluated the efficacy and renoprotection of JPBS therapy for the treatment of anemia in CKD (Wang, 2013; Li, 2016; Zhang, 2017). Therefore, in the current study, we performed a meta-analysis to assess JPBS therapy for patients with CKD anemia. The results reported here will help lay a foundation for the treatment of CKD anemia with JPBS therapy.

## Materials and Methods

### Database Searching

Seven different databases (i.e., PubMed, Cochrane Library, EMBASE, Wanfang Database, China National Knowledge Infrastructure, Chinese Science and Technique Journals Database, and China Biological Medicine Database) were independently searched from inception to April 2019 (by authors YZ and QX). The search terms used were: (jianpi OR bushen OR invigorating spleen OR reinforcing kidney) AND (renal anemia OR anemia of chronic kidney disease OR anemia of renal disease). These terms were translated into Chinese when searching the Chinese databases. Additional studies missed by the electronic search were also searched manually.

### Inclusion Criteria

The inclusion criteria were as follows: (1) randomized controlled trial (RCT); (2) patients diagnosed with CKD anemia according to the KDIGO Clinical Practice Guideline for Anemia in Chronic Kidney Disease (McDonald, 2012) and the Diagnosis and Treatment of Renal Anemia with Chinese Expert Consensus (Revision 2018) (Renal Physicians Association Branch of China Consensus Group of Experts on the Diagnosis and Treatment of Renal Anemia, 2018), with no limitations related to age, ethnicity, gender, or primary disease; (3) adoption of JPBS therapy combined with WM (iron supplements, ESAs) as the treatment group, with WM alone applied as the control group. Treatment needed to last at least eight weeks and the sample size needed to be ≥15; (4) primary outcomes to include clinical effective rate and hemoglobin (Hb) level, secondary outcomes to include serum ferritin (SF), red blood cell (RBC), hematocrit (HCT), serum creatinine (SCr), blood urea nitrogen (BUN) levels and number of adverse events; and (5) results available in either English or Chinese.

### Exclusion Criteria

The exclusion criteria were as follows: (1) non-randomized controlled trial; (2) study subjects did not meet the diagnostic criteria of renal anemia; (3) both treatment and control groups were treated with CHM only; and (4) studies were duplicated publications or articles with unavailable data.

### Quality Assessment and Data Extraction

The first author, year of publication, region, sample size, age, intervention, follow-up time, and main outcomes of each eligible study were collected by two authors (YZ and QX). Based on bias risk assessment of the Cochrane collaboration tool, the methodological quality of the selected studies was evaluated by YZ and QX. The assessed items included: (1) method of random allocation and allocation concealment; (2) blinding method of participants and personnel; (3) blinding method of outcome assessment; (4) integrity of data; (5) selectivity of result reporting; and (6) other biases. For any disagreement, the issue was resolved by discussion with a third author (CL).

### Data Analysis

RevMan v5.3 from Cochrane was employed to analyze data. For dichotomous outcomes, risk ratio (RR) with a 95% confidence interval (CI) was used. For continuous outcomes, mean difference (MD) with a 95% confidence interval (CI) was adopted. Data heterogeneity was assessed by χ^2^ and *I* ^2^ tests. If heterogeneity existed in pooled studies (*I* ^2^ ≥ 50%), a random model was applied; if not, a fixed model was applied. Statistically significant differences were considered at *P* < 0.05. Publication bias was evaluated by funnel plots and calculated using Begg’s/Egger’s tests through STATA v15.0. Sensitivity analysis was conducted by removing individual studies to assess the stability of the results.

## Results

### Search Results and Study Characteristics

**Figure 1:**
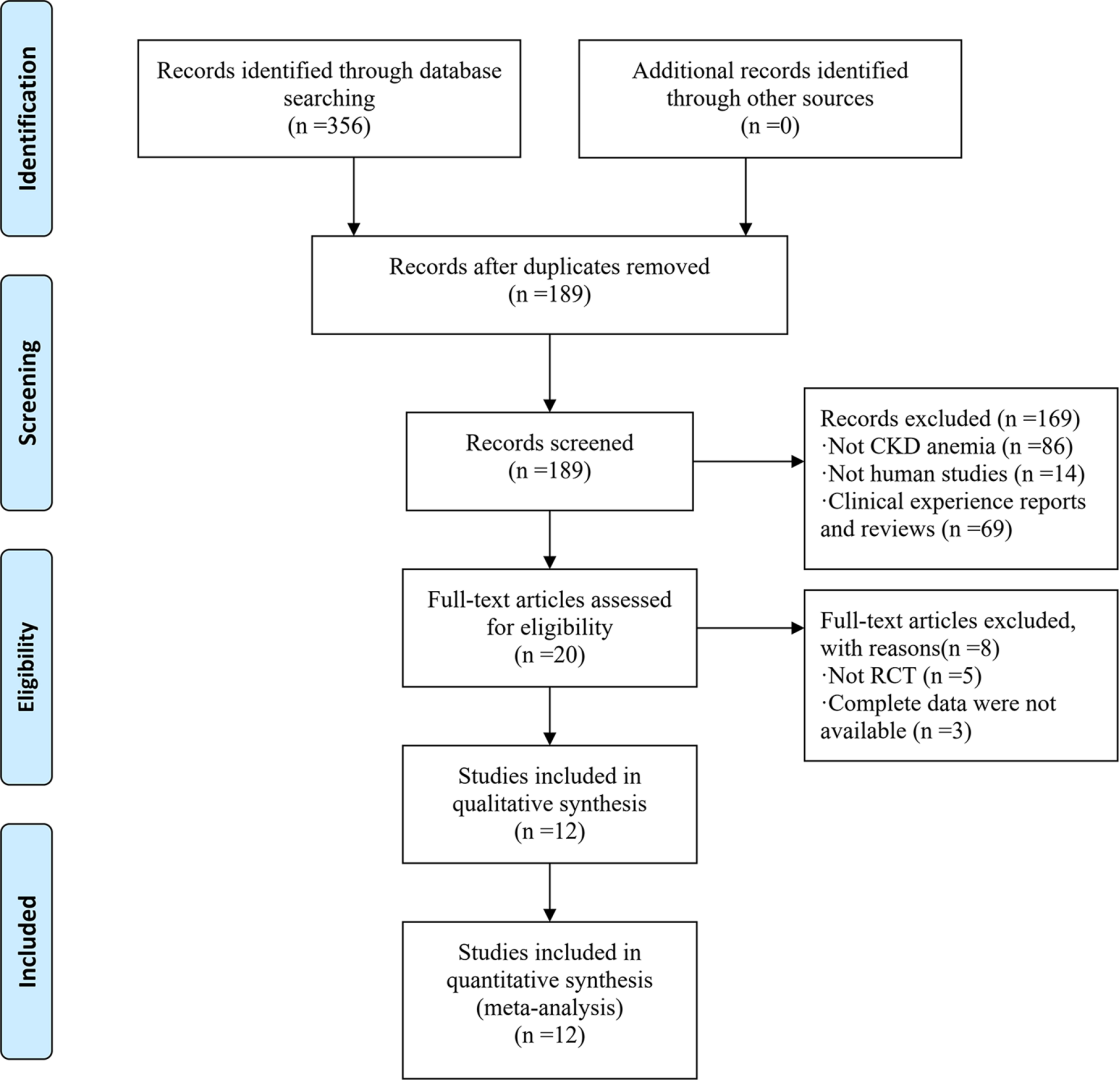
Flow chart of study selection process.

**Figure 1** shows a flow diagram of the study. Based on our database search, 356 articles reporting on CKD anemia treatment using JPBS therapy were identified. Of these articles, 167 duplicated publications were excluded. After going through the titles and abstracts, another 169 articles were excluded due to non-CKD anemia, non-human studies, and clinical experience reports and reviews. The 20 remaining full-text articles were further screened, with another eight eliminated because of non-RCT or incomplete data. Finally, a total of 12 articles with 799 patients were enrolled in this meta-analysis (Li et al., 2003; Cheng et al., 2005; Bao et al., 2008; Zhou et al., 2008; Liang, 2009; Qu, 2011; Wang, 2012; Liu, 2013; Wang, 2013; Li, 2016; Du, 2017; Zhang, 2017). Among them, 418 patients received JPBS therapy and 381 patients received WM treatment. The main characteristics of the enrolled articles are presented in **Table 1**.

**Table 1:** Characteristics of RCTs included in the study.

Study ID	Region	Sample size (T/C)	Age (y)		Gender (M/F)		Intervention		Duration(weeks)	Outcome
			T	C	T	C	T	C		
Wang, 2012	China	40/40	51.6	53.4	17/23	19/21	JPBS+WM	WM	8	a,b,e,f,g
Du, 2017	China	15/15	49.28 ± 8.24	50.15 ± 5.26	8/7	8/7	JPBS+WM	WM	8	b,d,e,f,g
Li et al., 2003	China	30/30	45.8 ± 19.9	47.5 ± 13.6	21/9	20/10	JPBS+WM	WM	12	a,b,e,f,g
Qu, 2011	China	27/25	NA	NA	NA	NA	JPBS+WM	WM	8	b,d,e,f,g
Zhang, 2017	China	30/30	NA	NA	17/13	12/18	JPBS+WM	WM	12	a,b,c,e,f,g
Liu, 2013	China	27/27	52.70 ± 15.4	42.66 ± 13.8	17/10	15/12	JPBS+WM	WM	8	a,b,e,f
Li, 2016	China	60/60	44.6 ± 12.7	44.3 ± 12.5	38/22	40/20	JPBS+WM	WM	12	a,b,c,d,e,f,g
Bao et al., 2008	China	34/27	47.5 ± 10.4	46.8 ± 11.5	18/16	15/12	JPBS+WM	WM	8	a,b,e,f,g
Wang, 2013	China	31/31	NA	NA	14/17	16/15	JPBS+WM	WM	8	a,b,d,e,f,g
Cheng et al., 2005	China	30/30	47.8 ± 14.5	46.3 ± 12.4	14/16	15/15	JPBS+WM	WM	8	a,b,d,e,f,g
Zhou et al., 2008	China	56/30	44.21 ± 12.2	44.25 ± 11.2	30/26	18/12	JPBS+WM	WM	12	a,b,e,f
Liang, 2009	China	38/36	NA	NA	NA	NA	JPBS+WM	WM	8	a,b,e,f,g

T, treatment group; C, control group; NA, not available; M/F, male/female; CHM, Chinese herbal medicine; JPBS, Jianpi Bushen; WM, western medicine; outcomes: a, clinical efficacy; b, Hb; c, SF; d, RBC; e, HCT; f, SCr; g, BUN.

### Risk of Bias

**Figure 2:**
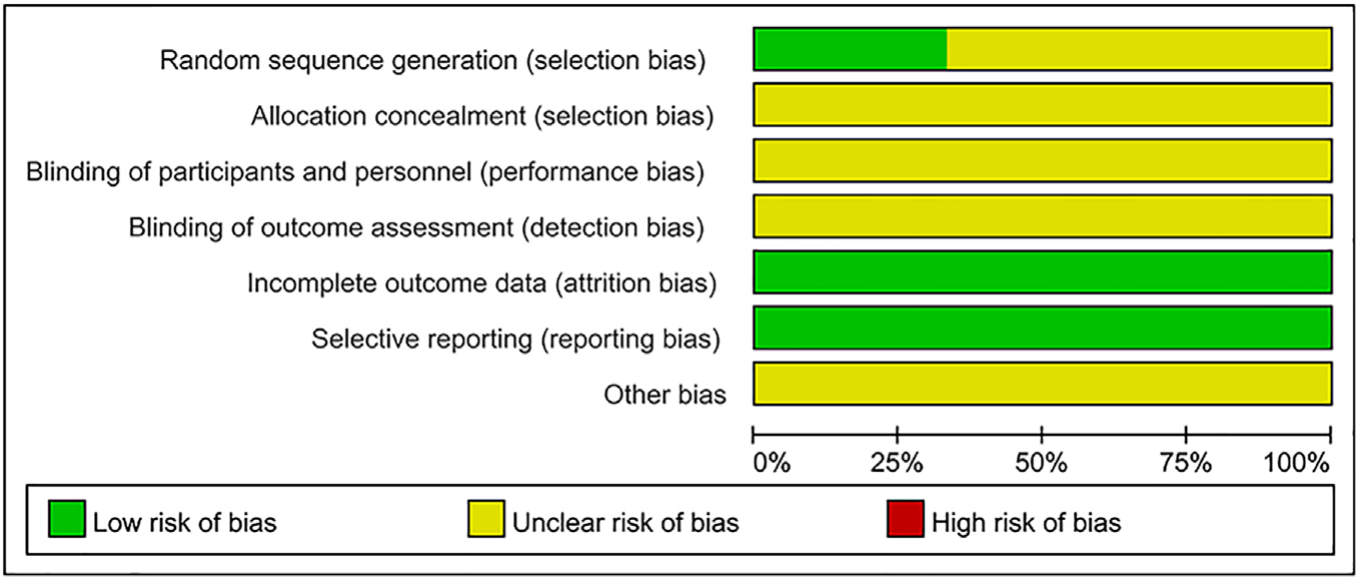
Risk of bias graph.

**Figure 3:**
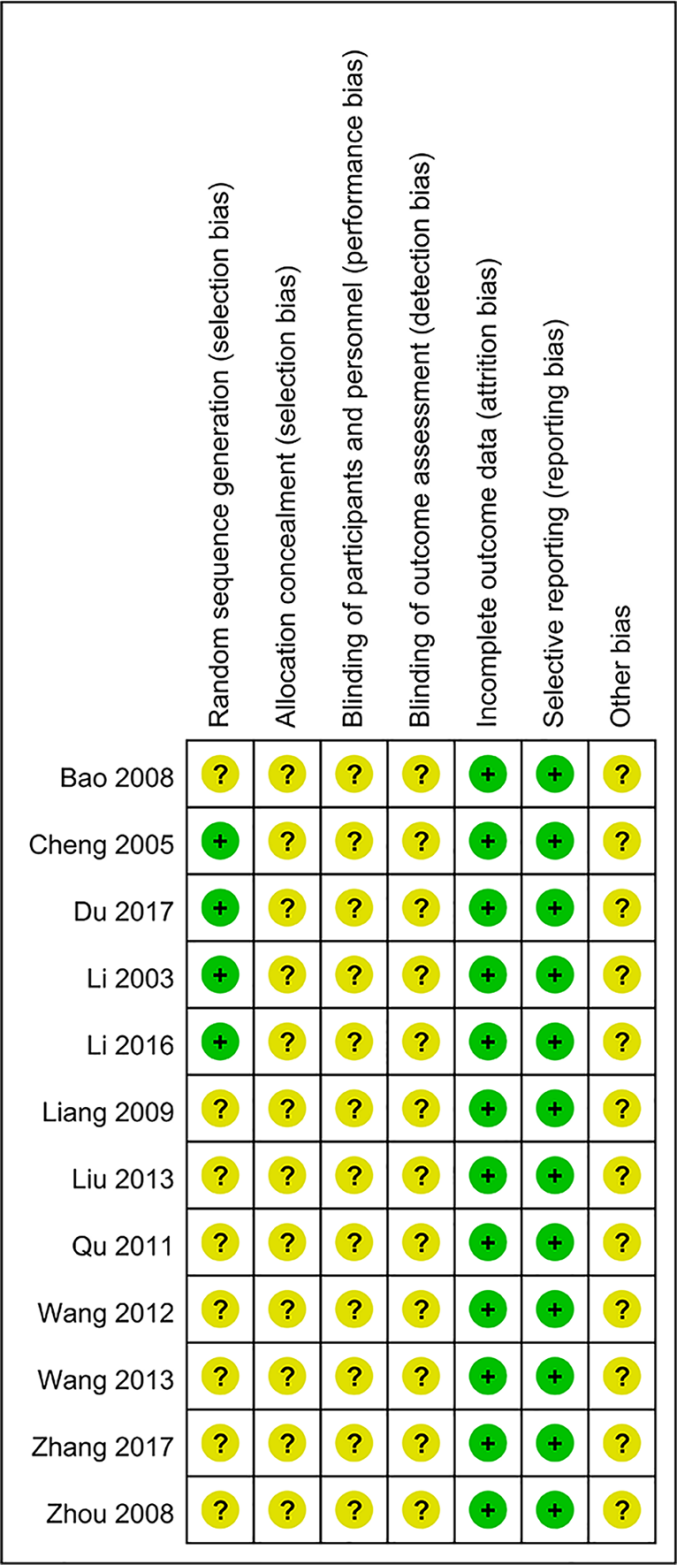
Risk of bias summary.

As shown in **Figures 2** and **3**, the methodological quality of the included articles was relatively low. All enrolled articles claimed to be randomized, but only four described their random methods (Li et al., 2003; Cheng et al., 2005; Li, 2016; Du, 2017). No article mentioned allocation concealment or blinding methods. All articles had complete data, and there was no selective reporting or other biases.

### Effects of Intervention

#### Clinical Efficacy Rate

**Figure 4:**
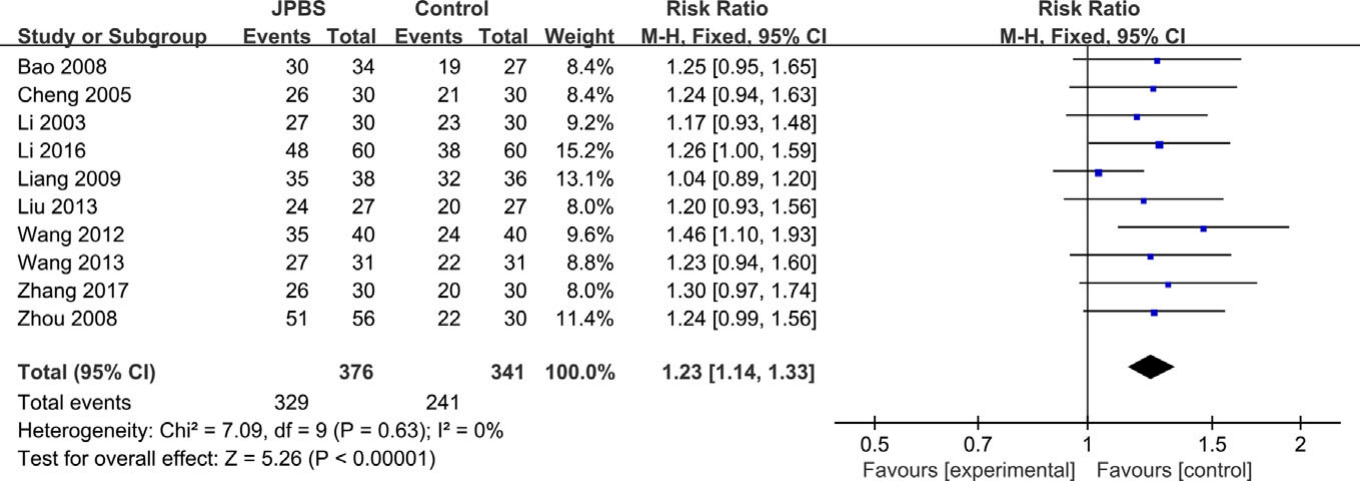
Forest plot of clinical efficacy rate.

Clinical efficacy rates were reported in 10 studies (Li et al., 2003; Cheng et al., 2005; Bao et al., 2008; Zhou et al., 2008; Liang, 2009; Wang, 2012; Liu, 2013; Wang, 2013; Li, 2016; Zhang, 2017), totaling 717 patients (376 cases in the JPBS therapy group and 341 cases in the WM treatment group). Results indicated that the clinical efficacy rates of the JPBS therapy group were higher than those of the WM treatment group [RR = 1.23, 95% CI: (1.14, 1.33), *P* < 0.00001, **Figure 4**].

#### Hb Levels

**Figure 5:**
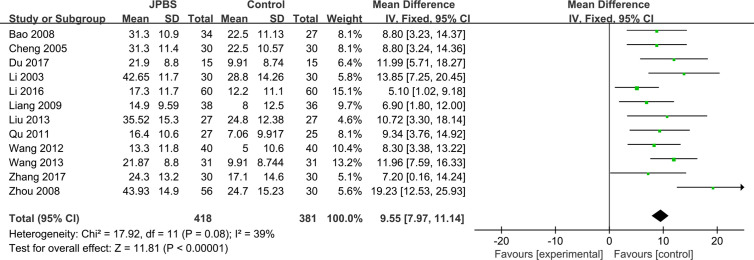
Forest plot of Hb levels.

Hb data were provided in all studies (Li et al., 2003; Cheng et al., 2005; Bao et al., 2008; Zhou et al., 2008; Liang, 2009; Qu, 2011; Wang, 2012; Liu, 2013; Wang, 2013; Li, 2016; Du, 2017; Zhang, 2017), totaling 799 patients (418 cases in the JPBS therapy group and 381 cases in the WM treatment group). As data heterogeneity was not found (*I* ^2^ = 39%, *P* = 0.08), the fixed effect model was applied (**Figure 5**). Results indicated that the increase in serum Hb levels was greater in the JPBS therapy group than in the WM treatment group [WMD = 9.55, 95% CI: (7.97, 11.14), *P* < 0.00001].

#### SF Levels

**Figure 6:**
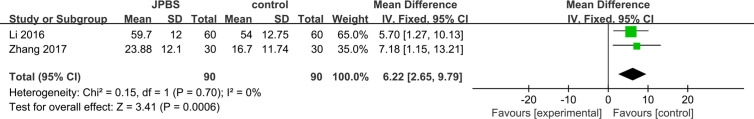
Forest plot of SF levels.

SF data were provided in two studies (Li, 2016; Zhang, 2017), totaling 180 patients (90 cases in the JPBS therapy group and 90 cases in the WM treatment group). As data heterogeneity was not found (*I* ^2^ = 0%, *P* = 0.70), the fixed effect model was applied (**Figure 6**). Results indicated that the increase in serum SF levels was greater in the JPBS therapy group than in the WM treatment group [WMD = 6.22, 95% CI: (2.65, 9.79), *P* = 0.0006].

#### RBC Levels

**Figure 7:**
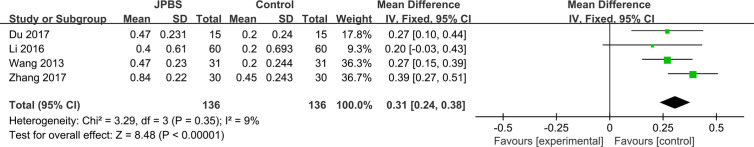
Forest plot of RBC levels.

RBC data were provided in four studies, totaling 272 patients (136 cases in the JPBS therapy group and 136 cases in the WM treatment group) (Wang, 2013; Li, 2016; Du, 2017; Zhang, 2017). No significant heterogeneity was found across the studies (*I* ^2^ = 9%, *P* = 0.35), and thus the fixed effect model was used (**Figure 7**). Results indicated that the increase in serum RBC levels was greater in the JPBS therapy group than in the WM treatment group [WMD = 0.31, 95% CI: (0.24, 0.38), *P* < 0.00001].

#### HCT Levels

**Figure 8:**
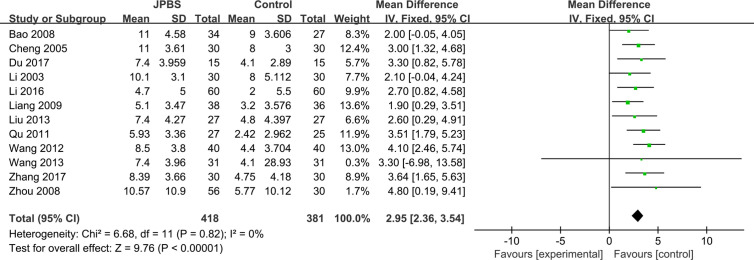
Forest plot of HCT levels.

HCT data were provided in all included studies, totaling 799 patients (418 cases in the JPBS therapy group and 381 cases in the WM treatment group) (Li et al., 2003; Cheng et al., 2005; Bao et al., 2008; Zhou et al., 2008; Liang, 2009; Qu, 2011; Wang, 2012; Liu, 2013; Wang, 2013; Li, 2016; Du, 2017; Zhang, 2017). The fixed effect model was applied as no data heterogeneity was found among the studies (*I* ^2^ = 0%, *P*= 0.82) (**Figure 8**). Compared with that in the WM treatment group, the level of serum HCT showed greater improvement in the JPBS therapy group (WMD = 2.95, 95% CI: (2.36, 3.54), *P* < 0.00001).

#### SCr Levels

**Figure 9:**
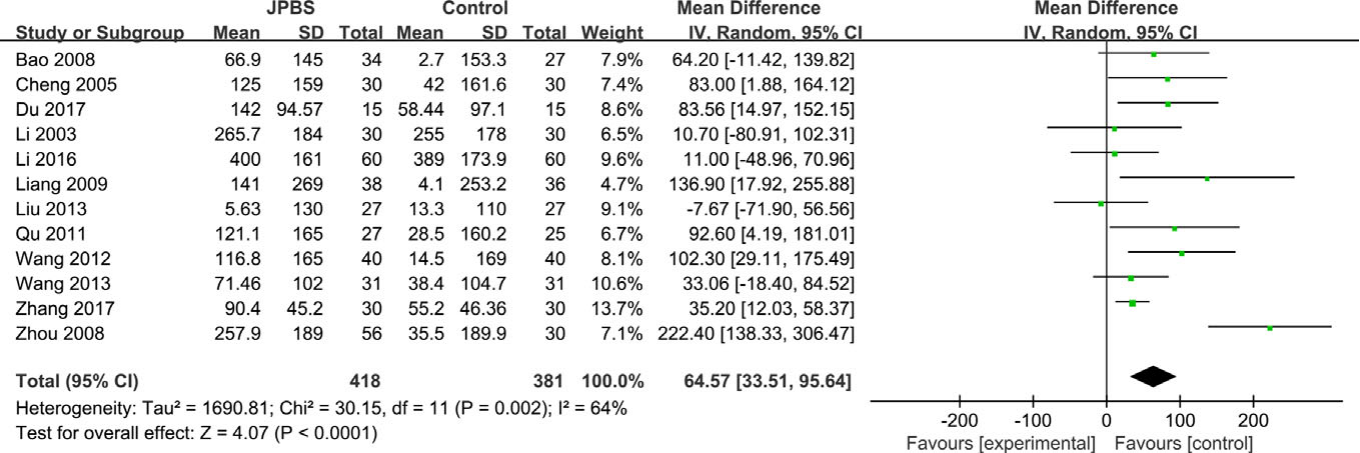
Forest plot of SCr levels.

SCr data were reported in all studies, totaling 799 patients (418 cases in the JPBS therapy group and 381 cases in the WM treatment group) (Li et al., 2003; Cheng et al., 2005; Bao et al., 2008; Zhou et al., 2008; Liang, 2009; Qu, 2011; Wang, 2012; Liu, 2013; Wang, 2013; Li, 2016; Du, 2017; Zhang, 2017). Data heterogeneity was found among the studies (*I* ^2^ = 64%, *P* = 0.002), and thus we applied the random effect model (**Figure 9**). Compared with that in the WM treatment group, the level of serum SCr showed greater improvement in the JPBS therapy group (WMD = 64.57, 95% CI: (33.51, 95.64), *P* < 0.0001).

#### BUN Levels

**Figure 10:**
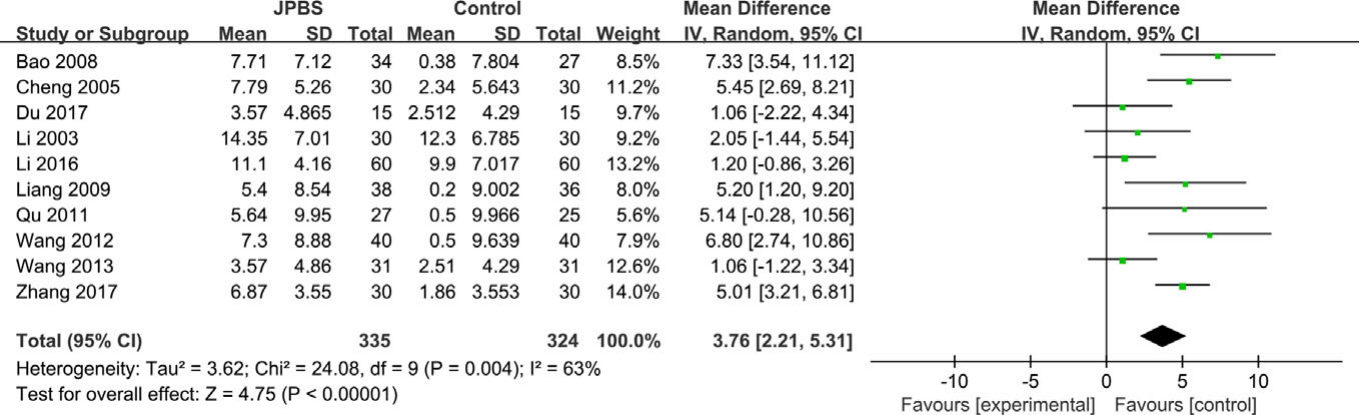
Forest plot of BUN levels.

BUN data were reported in 10 studies, totaling 659 patients (335 cases in the JPBS therapy group and 324 cases in the WM treatment group) (Li et al., 2003; Cheng et al., 2005; Bao et al., 2008; Liang, 2009; Qu, 2011; Wang, 2012; Wang, 2013; Li, 2016; Du, 2017; Zhang, 2017). Based on data heterogeneity analysis (*I* ^2^ = 63%, *P* = 0.004), the random effect model was adopted (**Figure 10**). Results showed that the level of serum BUN decreased in the JPBS therapy group compared with that in the WM treatment group [WMD = 3.76, 95% CI: (2.21, 5.31), *P* < 0.00001].

### Adverse Event Reporting

Two studies reported adverse events. Liang (2009) reported six cases of high blood pressure in the control group. Li (2016) reported seven cases of high blood pressure in the JPBS therapy group (adverse event frequency of 7/60), as well as one case of allergic reaction and eight cases of high blood pressure in the control group (adverse event frequency of 9/60). The Renal Association Clinical Practice Guideline on Anemia of Chronic Kidney Disease suggests there may be an increase in the risk of allergic reaction during intravenous iron injection and of hypertension with ESA administration (Mikhail et al., 2017). Thus, the adverse events reported above may be caused by WM treatment. Based on the adverse events reported in the selected studies, JPBS therapy appears to be safe and does not show an increase in adverse reactions compared with WM alone.

### Sensitivity Analysis

**Figure 11:**
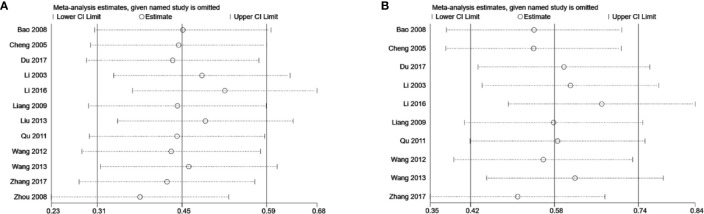
Sensitivity analysis plots of **(A)** SCr and **(B)** BUN.

Due to the considerable heterogeneity in the SCr and BUN results found among the studies, sensitivity analysis was carried out to investigate the source of this data heterogeneity. Sensitivity analysis suggested that exclusion of any one study for each outcome did not alter the overall results, indicating that the conclusions were robust (**Figure 11**).

### Publication Bias

**Figure 12:**
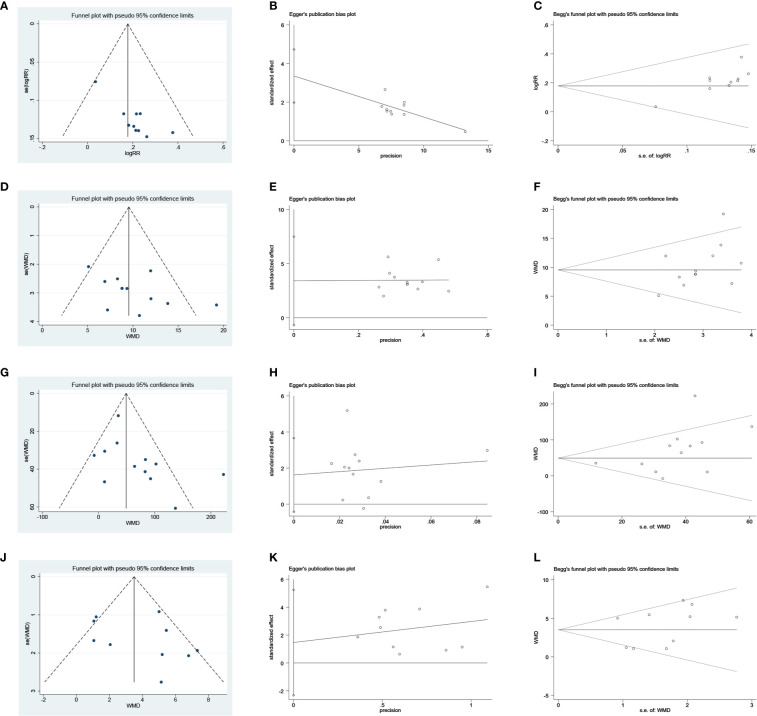
Publication bias plots. **(A)** Funnel plot of clinical efficacy rate; **(B)** Egger’s plot of clinical efficacy rate; **(C)** Begg’s plot of clinical efficacy rate; **(D)** Funnel plot of Hb; **(E)** Egger’s plot of Hb; **(F)** Begg’s plot of Hb; **(G)** Funnel plot of SCr; **(H)** Egger’s plot of SCr**; (I)** Begg’s plot of SCr; **(J)** Funnel plot of BUN; **(K)** Egger’s plot of BUN; **(L)** Begg’s plot of BUN.

Publication bias was assessed based on the clinical efficacy and Hb, SCr, and BUN results. The clinical efficacy funnel plot revealed a slight asymmetry and Egger’s (*t* = 5.61, *P* = 0.001) and Begg’s tests (*z* = 2.15, *P* = 0.032) indicated possible publication bias (**Figures 12A–C**). Visual assessment of funnel plots and Egger’s (Hb: *t* = 1.86, *P* = 0.093; SCr: *t* = 1.77, *P* = 0.107; BUN: *t* = 0.89, *P* = 0.398) and Begg’s test results (Hb: *z* = 1.85, *P* = 0.064; SCr: *z* = 1.85, *P* = 0.064; BUN: *z* = 0.89, *P* = 0.371) showed no publication bias for Hb, SCr, and BUN (**Figures 12D–L**).

## Discussion

Anemia is a common and serious complication for millions of patients with CKD (Locatelli et al., 2013). However, debate continues about the best strategies for the management of CKD anemia; for example, Solomon et al. (2010) found that treatment of CKD anemia with ESAs is inferior to that using placebo. In this context, current treatment of CKD anemic patients only reduces the risk of blood transfusion, rather than managing anemia and its associated risks. Nowadays, CHM is frequently applied to improve the symptoms and signs associated with CKD anemia. In accordance with TCM theory, the pathogenesis of CKD anemia is related to deficiencies of the spleen and kidney. Therefore, invigorating the spleen and reinforcing the kidney (Jianpi Bushen) are the main principles of TCM treatment for CKD anemia. In recent years, an increasing number of randomized controlled trials have been conducted using JPBS therapy for the treatment of CKD anemia, providing opportunities for objective and comprehensive evaluation of this method. Here, we performed a meta-analysis to clarify the efficacy of this treatment strategy.

The main findings of our meta-analysis were as follows: (1) compared with WM alone, JPBS therapy combined with WM improved the clinical efficacy rate; (2) compared with WM alone, JPBS therapy combined with WM significantly improved serum Hb, SF, RBC, and HCT levels in patients suffering CKD anemia; (3) adjunctive treatment with JPBS therapy decreased serum SCr and BUN levels in CKD anemic patients; and (4) JPBS therapy did not increase adverse reactions compared with WM alone. These results suggest that JPBS therapy may have beneficial effects in the management of CKD anemic patients.

The significant improvements in clinical efficacy rate and Hb, SF, RBC, HCT, SCr, BUN levels may be attributed to the promotion of hemoglobin formation and the renoprotective effects of CHM. In the included studies, the most frequently used Chinese medicines with the JPBS therapy were Astragali Radix, Angelicae Sinensis Radix, Codonopsis Radix, and Lycium Barbarum (**Supplementary Tables 1** and **2**). The main components of Astragali Radix, i.e., flavonoids and polysaccharides, are reported to promote erythroid differentiation, increase fetal hemoglobin synthesis, and regulate EPO expression (Yang et al., 2010; Zheng et al., 2011; Zhang et al., 2017). Angelicae Sinensis Radix polysaccharides are thought to exhibit an anti-anemic effect, which can restore EPO production and improve iron availability *via* stabilizing HIF2α protein and suppressing inflammation (Wang et al., 2018). Codonopsis Radix polysaccharides significantly elevate peripheral blood Hb levels in mice (Gao et al., 2018). In respect of renoprotective effects, Zhao et al. (2016) reported that Lycium Barbarum polysaccharides improve renal function and alleviate kidney inflammation in diabetic rabbits. Therefore, a combination of these CHMs could theoretically alleviate anemia and improve renal function, with a clear therapeutic effect on CDK anemia.

The results of the present study are consistent with research from Li et al. (2004). However, our study varies from previous published meta-analysis related to CKD anemia. For example, Zhao et al. (2017) suggested that Danggui Buxue Decoction combined with WM may be more effective in the treatment of CKD anemia than WM alone. However, they did not specifically reveal the TCM pathogenesis of CKD anemia and JPBS therapy. Based on another meta-analysis, Elliott et al. (2017) reported that ESAs may not have robust or reproducible benefits on renal function in humans. In our meta-analysis, JPBS therapy combined with WM showed positive effects on renal function. However, there are several limitations to this study. First, all included studies were conducted in China, which may limit the wide application of the results. Second, the methodological quality of the enrolled articles was generally low, with none providing a description of allocation concealment or blinding method, which may affect the credibility of the results. Third, there was a potential publication bias in regard to the clinical efficacy rate, which may be due to the flawed research design of small studies or the lack of publication of small studies with negative results. Lastly, data heterogeneity among the studies, including for partial results, was also significant, although sensitivity analysis indicated that our results were stable and reliable. The heterogeneity among the studies may be due to differences in treatment dose, intervention duration, and primary disease of CKD.

Our study demonstrated that JPBS therapy shows good potential for the treatment of CKD anemia, with the possibility of alleviating anemia while improving renal function. However, further research is needed to explore the possible mechanisms of JPBS therapy in the treatment of CKD anemia.

## Conclusion

The results of this study indicate that JPBS therapy combined with WM is more effective than WM alone in terms of inducing remission in CKD anemia, especially in regard to clinical efficacy rates and improvement in Hb, SF, RBC, HCT, SCr, and BUN levels. However, our conclusions should be interpreted with some caution due to the relatively poor quality of the included studies. Therefore, to determine the clinical value of JPBS therapy in the treatment of CKD anemia, further standard, double-blind, randomized studies are needed.

## Author Contributions

LL and YB designed the study. YZ, QX, and CL contributed to the data collection and data analysis. Results were interpreted by ZW and XZ. LL drafted the original manuscript. All authors contributed to the article and approved the submitted version.

## Funding

This study was supported by Academic Experience Inheritance of the Sixth National Group of Old Chinese Medicine Experts of the State Administration of Traditional Chinese Medicine [No. 2017 (29)].

## Conflict of Interest

The authors declare that the research was conducted in the absence of any commercial or financial relationships that could be construed as a potential conflict of interest.
