# Supplementary material for: Effects of Jianpi Bushen Therapy for Treatment of CKD Anemia: A Meta-Analysis of Randomized Controlled Trials
Source: Front Pharmacol. 2020 Sep 15;11:560920. doi: 10.3389/fphar.2020.560920 (PMC7523512; doi:10.3389/fphar.2020.560920)
Supplement: Supplementary file 1 [file Table_1.docx]

**SUPPLEMENTARY TABLE** **1** Ingredients and preparation quality control of JPBS therapy in included studies.

| **Study ID** | **Prescription name** | **Species, concentration** | **Preparations** | **Quality control** | **Chemical analysis reported?(Y/N)** |
| --- | --- | --- | --- | --- | --- |
| Wang, 2012 | Jianpi Yishen Huazhuo Decoction | Astragali Radix,30g; Angelicae Sinensis Radix,10g; Codonopsis Radix,15g;  Coicis Semen,15g; PolygoniMultiflori Radix,15g; Rehmanniae radix,15g; AsiniCoriiColla,10g; Lycium Barbarum,15g; Paeoniae Radix Alba,10g; Chuanxiong Rhizoma,10g; Radix Salviae,15g; Placenta Hominis,6g; Citrus Reticulata,10g; Arum Ternatum Thunb,10g; Colla Cornus Cervi,10g; Radix Rhei Et Rhizome,6g | Decoction | Hospital preparation | N |
| Du, 2017 | Bupi Qiangshen Shengxue Decoction | Ginseng Radix,30g; Astragali Radix,30g; Atractylodis Macrocephalae Rhizoma,20g; Dioscorea Opposita,30g; Lycium Barbarum,20g; RehmanniaeRadix,15g; Citrus Reticulata,15g; Angelicae Sinensis Radix,15g; Cuscutae Semen,15g; Poria,15g; Licorice,10g | Decoction | Hospital preparation | N |
| Li et al., 2003 | Bushen Jianpi Formula | Ginseng Radix; Astragali Radix; LigustriLucidi Fructus; EcliptaeHerba; Lycium Barbarum; Angelicae Sinensis Radix; Radix Rhei Et Rhizome | Decoction | Hospital preparation | N |
| Qu, 2011 | Jianpi Bushen Formula | Astragali Radix; Codonopsis Radix; Angelicae Sinensis Radix; Atractylodis Macrocephalae Rhizoma; Poria; PolygoniMultiflori Radix; CistanchesHerba; Cuscutae Semen; Halloysitum Rubrum; Forsythiae Fructus; Arum Ternatum Thunb; LeonuriHerba; Radix Salviae | Decoction | Hospital preparation | N |
| Zhang, 2017 | Yishen Jianpi Huazhuo Formula | Astragali Radix,20g; Codonopsis Radix,15g; Rehmanniae radix,15g; Angelicae Sinensis Radix,15g; Dioscoreae Rhizoma,15g; Atractylodis Macrocephalae Rhizoma,15g; Corni Fructus,15g; Lycium Barbarum,20g; Poria,20g; Amomum Aurantiacum,10g; Eupatorium Fortunei Turcz,15g; Coicis Semen,15g; Licorice,10g | Decoction | Hospital preparation | N |
| Liu, 2013 | Yishen Jianpi Decoction | Astragali Radix,30g; RehmanniaeRadix,20g; Codonopsis Radix,20g; Atractylodis Macrocephalae Rhizoma,15g; Poria,15g; Paeoniae Radix Alba,15g; Lycium Barbarum,15g; Cuscutae Semen,15g; Radix Salviae,30g; Angelicae Sinensis Radix,20g; Licorice,6g | Decoction | Hospital preparation | N |
| Li, 2016 | Wenshen Bupi Shengxue Paste | Cuscutae Semen,400g; CistanchesHerba,400g; Morindae Officinalis Radix,400g; Atractylodis Macrocephalae Rhizoma,400g; Poria,300g; Codonopsis Radix,300g; Astragali Radix,600g; Angelicae Sinensis Radix,400g; ZiziphiSpinosae Semen,300g; Licorice,100g; Citrus Reticulata,300g; Arum Ternatum Thunb,300g; Amomum Aurantiacum,100g | Paste | Hospital preparation | N |
| Bao et al., 2008 | Jianpi Yishen Xiezhuo Formula | Codonopsis Radix,30g; Radix Salviae,30g; Astragali Radix,30g; Lycium Barbarum,20g; Angelicae Sinensis Radix,15g; Poria,15g; Atractylodis Macrocephalae Rhizoma,15g; CistanchesHerba,15g; Chuanxiong Rhizoma,10g; Arum Ternatum Thunb,10g; Perillae Folium,10g; Radix Rhei Et Rhizome,10g; Amomum Aurantiacum,4g | Decoction | Hospital preparation | N |
| Wang, 2013 | Jianpi Yishen Shengxue Formula | Astragali Radix,60g; Codonopsis Radix,30g; Atractylodis Macrocephalae Rhizoma,20g; Dioscoreae Rhizoma,30g; Lycium Barbarum,15g; RehmanniaeRadix,15g; Angelicae Sinensis Radix,15g; Cuscutae Semen,15g; Citrus Reticulata,15g; Poria,15g; Licorice,10g | Decoction | Hospital preparation | N |
| Cheng et al., 2005 | Jianpi Bushen Xiezhuo Formula | HedyotisDiffusaeHerba,30g; Astragali Radix,20g; Radix Salviae,20g; LeonuriHerba,20g; Rehmanniae Radix,10g; Angelicae Sinensis Radix,10g; Poria,10g; LigustriLucidi Fructus,10g; Radix Rhei Et Rhizome,10g | Decoction | Hospital preparation | N |
| Zhou et al., 2008 | Bushen Jianpi Formula | Codonopsis Radix,30g; Astragali Radix,30g; LigustriLucidi Fructus,20g; EcliptaeHerba,20g; Angelicae Sinensis Radix,20g; Lycium Barbarum,15g; Radix Rhei Et Rhizome,6g | Decoction | Hospital preparation | N |
| Liang, 2009 | Bupi Yishen Shengxue Formula | Codonopsis Radix,15g; Astragali Radix,15g; Coicis Semen,15g; Angelicae Sinensis Radix,12g; RehmanniaeRadix,12g; EpimrdiiHerba,12g; Lycium Barbarum,12g; Spatholobus Suberectus Dunn,25g; Radix Rhei Et Rhizome,5g | Decoction | Hospital preparation | N |
