# Supplementary material for: Effects of Jianpi Bushen Therapy for Treatment of CKD Anemia: A Meta-Analysis of Randomized Controlled Trials
Source: Front Pharmacol. 2020 Sep 15;11:560920. doi: 10.3389/fphar.2020.560920 (PMC7523512; doi:10.3389/fphar.2020.560920)
Supplement: Supplementary file 2 [file Table_2.docx]

**SUPPLEMENTARY TABLE** **2** Most commonly used ingredients in JPBS therapy.

| **Chinese name** | **Pharmaceutical name** | **Species** | **Family** | **N/12(%)** |
| --- | --- | --- | --- | --- |
| Huangqi | Astragali Radix | *Astragalus membranaceus* (Fisch.) Bge | Leguminosae | 12(100%) |
| Danggui | Angelicae Sinensis Radix | *Angelica sinensis* (Oliv.) Diels | Umbelliferae | 12(100%) |
| Dangshen | Codonopsis Radix | *Codonopsis pilosula* (Franch.) Nannf. | Campanulaceae | 9(75%) |
| Gouqizi | Lycium Barbarum | *Lycium barbarum* L. | Solanaceae | 9(75%) |
| Fuling | Poria | *Poria cocos* (Schw.) Wolf | Polyporaceae | 8(67%) |
| Baizhu | Atractylodis Macrocephalae Rhizoma | *Atractylodes macrocephala*Koidz. | Asteraceae | 7(58%) |
| Dahuang | Radix Rhei Et Rhizome | *Rheum officinale*Baill | Polygonaceae | 6(50%) |
| Dihuang | Rehmanniae radix | *Rehmannia glutinosa*Libosch | Scrophulariaceae | 6(50%) |
